# Multitask Learning with Convolutional Neural Networks and Vision Transformers Can Improve Outcome Prediction for Head and Neck Cancer Patients

**DOI:** 10.3390/cancers15194897

**Published:** 2023-10-09

**Authors:** Sebastian Starke, Alex Zwanenburg, Karoline Leger, Fabian Lohaus, Annett Linge, Goda Kalinauskaite, Inge Tinhofer, Nika Guberina, Maja Guberina, Panagiotis Balermpas, Jens von der Grün, Ute Ganswindt, Claus Belka, Jan C. Peeken, Stephanie E. Combs, Simon Boeke, Daniel Zips, Christian Richter, Esther G. C. Troost, Mechthild Krause, Michael Baumann, Steffen Löck

**Affiliations:** 1 Helmholtz-Zentrum Dresden–Rossendorf, Department of Information Services and Computing, 01328 Dresden, Germany; 2 OncoRay—National Center for Radiation Research in Oncology, Faculty of Medicine and University Hospital Carl Gustav Carus, Technische Universität Dresden, Helmholtz-Zentrum Dresden–Rossendorf, 01309 Dresden, Germany; alexander.zwanenburg@nct-dresden.de (A.Z.); karoline.leger@uniklinikum-dresden.de (K.L.); fabian.lohaus@uniklinikum-dresden.de (F.L.); annett.linge@uniklinikum-dresden.de (A.L.); christian.richter@oncoray.de (C.R.); esther.troost@uniklinikum-dresden.de (E.G.C.T.); mechthild.krause@uniklinikum-dresden.de (M.K.); michael.baumann@dkfz-heidelberg.de (M.B.); steffen.loeck@oncoray.de (S.L.); 3 German Cancer Research Center (DKFZ), Heidelberg and German Cancer Consortium (DKTK) Partner Site Dresden, 01309 Dresden, Germany; 4 National Center for Tumor Diseases (NCT), Partner Site Dresden, Germany: German Cancer Research Center (DKFZ), Heidelberg, Germany; Faculty of Medicine and University Hospital Carl Gustav Carus, Technische Universität Dresden, Dresden, Germany, and; Helmholtz Association/Helmholtz-Zentrum Dresden–Rossendorf (HZDR), 01307 Dresden, Germany; 5 Department of Radiotherapy and Radiation Oncology, Faculty of Medicine and University Hospital Carl Gustav Carus, Technische Universität Dresden, 01309 Dresden, Germany; 6 German Cancer Research Center (DKFZ), Heidelberg and German Cancer Consortium (DKTK) Partner Site Berlin, 10117 Berlin, Germany; goda.kalinauskaite@charite.de (G.K.); ingeborg.tinhofer@charite.de (I.T.); 7 Department of Radiooncology and Radiotherapy, Charité University Hospital, 10117 Berlin, Germany; 8 German Cancer Research Center (DKFZ), Heidelberg and German Cancer Consortium (DKTK) Partner Site Essen, 45147 Essen, Germanymaja.guberina@uk-essen.de (M.G.); 9 Department of Radiotherapy, Medical Faculty, University of Duisburg-Essen, 45147 Essen, Germany; 10 German Cancer Research Center (DKFZ), Heidelberg and German Cancer Consortium (DKTK) Partner Site Frankfurt, 60596 Frankfurt, Germany; panagiotis.balermpas@usz.ch (P.B.); jens.vondergruen@kgu.de (J.v.d.G.); 11 Department of Radiotherapy and Oncology, Goethe-University Frankfurt, 60596 Frankfurt, Germany; 12 German Cancer Research Center (DKFZ), Heidelberg and German Cancer Consortium (DKTK) Partner Site Munich, 80336 Munich, Germany; ute.ganswindt@i-med.ac.at (U.G.); claus.belka@med.uni-muenchen.de (C.B.); jan.peeken@tum.de (J.C.P.); stephanie.combs@tum.de (S.E.C.); 13 Department of Radiation Oncology, Ludwig-Maximilians-Universität, 80336 Munich, Germany; 14 Clinical Cooperation Group, Personalized Radiotherapy in Head and Neck Cancer, Helmholtz Zentrum Munich, 85764 Neuherberg, Germany; 15 Department of Radiation Oncology, Medical University of Innsbruck, Anichstraße 35, A-6020 Innsbruck, Austria; 16 Department of Radiation Oncology, Technische Universität München, 81675 Munich, Germany; 17 Institute of Radiation Medicine (IRM), Helmholtz Zentrum München, 85764 Neuherberg, Germany; 18 German Cancer Research Center (DKFZ), Heidelberg and German Cancer Consortium (DKTK) Partner Site Tübingen, 72076 Tübingen, Germany; simon.boeke@med.uni-tuebingen.de (S.B.); daniel.zips@med.uni-tuebingen.de (D.Z.); 19 Department of Radiation Oncology, Faculty of Medicine and University Hospital Tübingen, Eberhard Karls Universität Tübingen, 72076 Tübingen, Germany; 20 Helmholtz-Zentrum Dresden–Rossendorf, Institute of Radiooncology—OncoRay, 01328 Dresden, Germany; 21 German Cancer Research Center (DKFZ), Division Radiooncology/Radiobiology, 69120 Heidelberg, Germany; 22 German Cancer Consortium (DKTK), Core Center DKFZ, 69120 Heidelberg, Germany

**Keywords:** survival analysis, vision transformer, convolutional neural network, multitask learning, tumor segmentation, head and neck cancer, Cox proportional hazards, loco-regional control, progression-free survival, discrete-time survival models

## Abstract

**Simple Summary:**

Increasing treatment efficacy for head and neck cancer requires the utilization of patient-specific biomarkers to personalize therapy. Deep neural networks show promise for extracting prognostic biomarkers from medical imaging data to predict loco-regional tumor recurrence or disease progression. However, training these networks can be challenging due to limited available data, potentially affecting prediction quality. To address these challenges, we investigated the effectiveness of multiple multitask learning strategies, where two distinct outcome tasks and an auxiliary tumor segmentation objective were simultaneously optimized. This approach aimed to enhance the training process, leading to better parameter configurations and improved predictive performance. Our analysis, conducted on two multicentric datasets using convolutional neural networks and vision transformers, indicated performance benefits of outcome models trained using multitask strategies over models trained solely on a single outcome task.

**Abstract:**

Neural-network-based outcome predictions may enable further treatment personalization of patients with head and neck cancer. The development of neural networks can prove challenging when a limited number of cases is available. Therefore, we investigated whether multitask learning strategies, implemented through the simultaneous optimization of two distinct outcome objectives (multi-outcome) and combined with a tumor segmentation task, can lead to improved performance of convolutional neural networks (CNNs) and vision transformers (ViTs). Model training was conducted on two distinct multicenter datasets for the endpoints loco-regional control (LRC) and progression-free survival (PFS), respectively. The first dataset consisted of pre-treatment computed tomography (CT) imaging for 290 patients and the second dataset contained combined positron emission tomography (PET)/CT data of 224 patients. Discriminative performance was assessed by the concordance index (C-index). Risk stratification was evaluated using log-rank tests. Across both datasets, CNN and ViT model ensembles achieved similar results. Multitask approaches showed favorable performance in most investigations. Multi-outcome CNN models trained with segmentation loss were identified as the optimal strategy across cohorts. On the PET/CT dataset, an ensemble of multi-outcome CNNs trained with segmentation loss achieved the best discrimination (C-index: 0.29, 95% confidence interval (CI): 0.22–0.36) and successfully stratified patients into groups with low and high risk of disease progression (p=0.003). On the CT dataset, ensembles of multi-outcome CNNs and of single-outcome ViTs trained with segmentation loss performed best (C-index: 0.26 and 0.26, CI: 0.18–0.34 and 0.18–0.35, respectively), both with significant risk stratification for LRC in independent validation (p=0.002 and p=0.011). Further validation of the developed multitask-learning models is planned based on a prospective validation study, which has recently completed recruitment.

## 1. Introduction

Head and neck squamous cell carcinoma (HNSCC) is the sixth most-frequently occurring cancer type worldwide and poses a global threat for health, as incidences are rising and expected to exceed one million new cases per year by 2030 [1]. Cancers of the head and neck are frequently discovered only in the advanced stages. Despite multimodal treatments, consisting of a combination of surgery, chemo-, and radiotherapy, tumor responses remain heterogeneous and five-year survival rates can be as low as 50% for locally advanced cases [1,2,3]. Therefore, treatment individualization strategies based on patient-individual biomarkers should be considered for improving therapy outcomes [4]. Due to their widespread use for diagnosis, therapy planning, and patient follow-up, medical imaging data such as computed tomography (CT) and positron emission tomography (PET) are routinely available and have emerged as valuable, non-invasive sources of information. Quantitative image biomarkers are increasingly proposed for prognosticating treatment outcomes, giving rise to the radiomics research field [5,6,7,8,9,10,11,12,13,14]. Image biomarkers can either be pre-defined and computed in a traditional radiomics workflow or learned from the data using deep learning (DL) techniques that employ, e.g., convolutional neural networks (CNNs) [15,16] or vision transformers (ViTs) [17]. CNNs comprise sequential layers of convolutional filters with learnable weights, interleaved with nonlinear activation functions. Images are processed by sliding the filters over the input to produce feature maps which are then passed on to the next layer. ViTs, on the other hand, partition images into non-overlapping regions and map them into high-dimensional vector representations. The obtained vector representations are then further processed using fully connected layers. In contrast to CNNs, information exchange between non-neighboring image regions can be exploited within every layer using a self-attention mechanism [18]. While the traditional radiomics approach allows for better control over model features and the possibility to include domain knowledge, resulting in increased interpretability, it also demands precise tumor delineation to be performed by clinical experts, which is a time-consuming and error-prone process, limiting throughput during model training and inference. The DL approach is appealing because precise expert delineations may not be required, and tissue surrounding the tumor can be included for assessment. Often, tumor bounding boxes or centroids are enough for cropping the volumetric data to regions feasible for the application of neural networks. Nevertheless, current DL-based techniques lack guarantees that outcome predictions are indeed related to tumor characteristics and bear the risk that models learn to infer their predictions from image content unrelated to the tumor. Recent work aimed to solve this issue through the incorporation of an auxiliary segmentation objective, forcing the network to learn about tumor location and shape when making predictions related to an endpoint of interest [19,20]. In contrast to the traditional radiomics workflow, such DL approaches only require precise tumor delineations during model training, but not for inference. As recently shown by Baek et al. [21], features learned in tumor segmentation tasks contain relevant information for outcome prediction as well, indicating that both tasks could complement each other for improved model performance. Combining model objectives with auxiliary tasks is called multitask learning [22,23,24]. Using a variety of auxiliary tasks, initial studies have confirmed the added value of such approaches for medical image and outcome analysis ([25,26,27,28,29,30,31,32,33,34]).

The Cox proportional hazards (CPH) model [35,36] is a frequently used outcome prediction model for censored data. It models the influence of covariates on the hazard function under the assumption that their effect is time-invariant, resulting in hazard rates which are proportional over time for individual patients. The CPH model does not assume a specific distribution of event times and has recently been used in neural network-based applications as well [12,36,37,38,39,40]. The exact evaluation of its underlying likelihood function requires knowledge of all samples in the dataset, which is not particularly suitable for the batchwise training scheme of neural networks, potentially complicating successful model training. Based on the reported benefits of the multitask paradigm, the addition of further loss feedback to neural network-based outcome models might stabilize training and improve performance. Therefore, we implemented multi-outcome multitask models that additionally incorporated the discrete-time outcome model suggested by Gensheimer et al. [41], which has a loss function more compatible with batchwise training and allows individual patient survival functions to be directly predicted at user-defined timepoints without being limited to the proportional hazards assumption.

In this paper, we aimed to (1) validate whether training outcome models with an auxiliary segmentation loss leads to enhanced model performance, and (2) verify whether an extension of multitask training to the multi-outcome prediction task through the simultaneous optimization of two established outcome models might further improve prediction quality. Both questions were analyzed in detail for CNNs and ViTs, the two most commonly utilized types of neural networks for image analysis tasks. Moreover, to demonstrate the applicability of our proposed approaches beyond a single set of patients, all analyses were carried out for two suitably sized, distinct multicentric datasets that differed in the analyzed treatment endpoint, HNSCC characteristics (predominantly human papillomavirus (HPV) negative vs. HPV positive), and the available imaging modalities (CT vs. PET/CT imaging data).

## 2. Materials and Methods

### 2.1. Patient Cohorts

Our analyses were based on two distinct patient cohorts. The first multicentric cohort consisted of non-contrast enhanced pre-treatment CT imaging data from 290 patients diagnosed with locally advanced HNSCC and treated between 1999 and 2013, originating from one of six partner sites of the German Cancer Consortium Radiation Oncology Group (DKTK-ROG). Follow-up information was collected up to 2016 for some patients. It was described and analyzed in previous publications of our group [9,12,13] and its patient characteristics are shown in Supplementary Table S1. Corresponding image acquisition parameters are provided in Supplementary Table S2. For the remainder of the article, we will refer to this cohort as the DKTK cohort. All patients were treated by primary radiochemotherapy. Ethical approval for the multicenter retrospective analyses was obtained from the Ethics Committee at the Technische Universität Dresden, Germany (EK177042017). All analyses were carried out in accordance with the relevant guidelines and regulations. Informed consent was obtained from all patients. CT scans were provided in the ‘digital imaging and communications in medicine’ (DICOM) format with contours of the primary gross tumor volume (GTV) manually delineated (F.L. or K.L.) and reviewed (E.G.C.T.) by experienced radiation oncologists. Compared to the work by Starke et al. [12], one patient of the exploration cohort had to be discarded due to problems in evaluating the DICOM data with the current version of the image processing software. For this cohort, loco-regional tumor control (LRC), i.e., the time in months between the start of radiochemotherapy and local or regional tumor recurrence with previous objective loco-regional tumor response, was the analyzed treatment endpoint. Based on the different included sites, this cohort was split into an exploratory (205 patients) and an independent validation (85 patients) cohort. The corresponding Kaplan–Meier (KM) estimates for LRC are shown in Supplementary Figure S1. The median follow-up times of patients alive were 53 and 43 months for the exploratory and independent validation cohorts, respectively (Supplementary Table S1).

The second cohort was comprised of pre-treatment ^18^F-fluorodeoxyglucose (FDG)-PET/CT imaging data used for initial staging of 224 oropharyngeal carcinoma patients from five different centers located in Canada and France. It was originally made available for participants of the HECKTOR challenge 2021 and re-used as a subset of the challenge in 2022 with the goal of developing models for tumor segmentation and outcome prediction. More specifically, the challenge required prediction of the progression-free survival (PFS) endpoint, defined by the organizers as either a size increase in known lesions (change in T and/or N stage), the appearance of new lesions (change in N and/or M stage), or disease-related death for previously stable patients [42,43,44]. PFS data were originally provided in days and converted to months for our analyses. CT imaging was performed without a contrast agent. Patient characteristics are summarized in Supplementary Table S3. The scanner models and image acquisition details are provided by Andrearczyk et al. [42]. This dataset will be referred to as the HECKTOR2021 cohort throughout this manuscript. A separate dataset for independent validation remained unpublished and only available to the organizers to rank the submitted solutions during the course of the challenge.

### 2.2. Image Preprocessing

CT imaging data and GTV contours of the DKTK cohort were extracted from DICOM files and interpolated to an isotropic voxel size of 1 ${\mathrm{mm}}^{3}$ using cubic splines with the in-house-developed ‘medical image radiomics processor’ (MIRP) python package [45,46]. For the HECKTOR2021 patients, we followed the suggestions of the challenge organizers by converting the PET intensities to body-weight-corrected standard uptake values (SUV), registering with the CT volume and interpolating to an isotropic voxel spacing of 1 mm${}^{3}$ using B-spline interpolation before cropping to bounding box coordinates of size 144×144×144 mm${}^{3}$ that captured the delineated primary GTV. Interpolation of the segmentation masks was performed using nearest neighbor interpolation. The CT and the PET volumes were subsequently concatenated in the channel dimension. Further preprocessing steps were applied on the fly when iterating over the patient data during network training and inference. Those involved the cropping of input images around the tumor’s center of mass to voxel dimensions of size 48×64×64. Additionally, CT numbers were clipped to the range [−200,200] Hounsfield units and normalized to the range [0,1] individually for each patient for both datasets. PET-based SUVs were not further normalized.

### 2.3. Analysis Design

In our investigations, we trained and evaluated CNN and ViT models using a five-fold cross-validation (CV) approach, during which patients were partitioned into groups used for training and internal performance testing. Patient partitioning to CV folds was stratified based on the event status of the analyzed endpoint. Three CV repetitions were run in accordance with our previous study [12]. Model predictions obtained during CV were further averaged to obtain ensemble predictions [47]. For patients of the independent validation cohort of the DKTK data, model predictions of all 15 models were aggregated in the ensemble prediction. In contrast, for patients of the exploratory cohort, ‘training’ and ‘internal test’ ensemble predictions were computed by considering predictions only from the corresponding CV models, for which the patient was part of the training (12 models) or internal test split (three models), respectively. Similarly, for patients of the HECKTOR2021 cohort, ‘training’ and ‘internal test’ ensembles involved predictions from 12 and three models, respectively.

### 2.4. Neural Network Architectures

**Figure 1:**
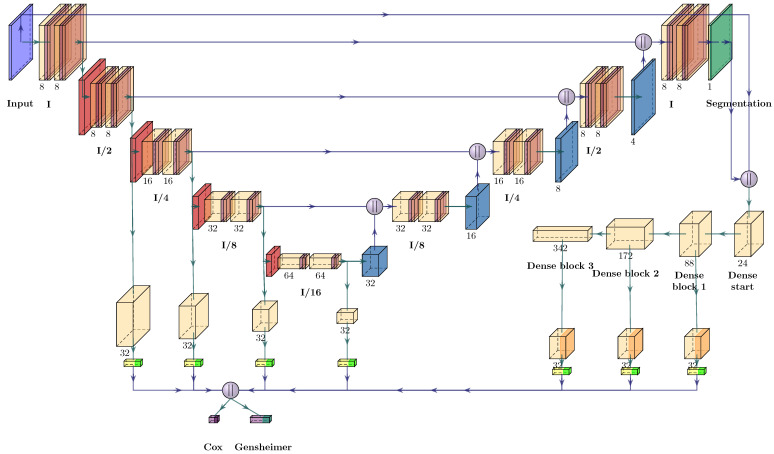
Architecture of the modified UNet used for simultaneous outcome prediction and segmentation. A sigmoid activation (dark green) is applied to the output of the UNet decoder to obtain segmentation predictions. For outcome prediction, convolutional layers (orange) with 32 filters of kernel size one are applied to the resulting feature maps of each encoder block, followed by a global average pooling (yellow), which reduces the spatial dimensions to a single number before being flattened (light green) into 32-dimensional vectors and concatenated. In the multi-outcome setting, the resulting 128-dimensional feature vector is passed in parallel to the two fully connected layers (purple) for the prediction of the log-hazard of the Cox model using a tanh activation and for the prediction of the conditional survival probabilities for the Gensheimer model, implemented using a sigmoid activation function. Optionally, a DenseNet comprised of three DenseNet blocks could be integrated into the model as well, which takes the image volume and the predicted segmentation mask as input. Additional features are then extracted from each DenseNet block by applying a convolutional layer with 32 filters and a ReLU activation function, before being pooled, flattened, and concatenated to the UNet encoder features.

Our investigated three-dimensional (3D) CNN architecture largely follows the design described by Meng et al. [20] and employs the structure of a UNet [48], as depicted in Figure 1. The encoder of our UNet consisted of five convolutional blocks, each comprised of two convolutional layers with a kernel size of three, and stride and padding of one, followed by instance normalization and the Leaky ReLU (α=0.1) activation function. After each block, the spatial resolution was halved using max pooling while the number of convolutional filters was doubled. Initially, eight filters were applied, which increased up to 64 filters at the bottom of the encoder. The decoder was designed to mirror the encoder design, with upsampling achieved using transposed convolutions with kernel sizes and strides of two. Upsampled representations were concatenated with the corresponding encoder feature maps. The UNet output consisted of a single channel. A sigmoid activation function was applied on each spatial location of the feature map for tumor segmentation. The latent feature maps of each encoder block were transformed by dedicated convolutional layers comprised of 32 filters with a kernel size of one, followed by an averaging operation over the spatial dimensions and flattening, resulting in 32 filters per encoder block. For the outcome prediction task, those representations were concatenated to a feature vector of 128 dimensions. Again following Meng et al. [20], a variant of the DenseNet architecture [49] was optionally used as a second network component to compute additional feature maps for the outcome prediction task. These were derived from the concatenation of the input image and the predicted segmentation mask to make explicit use of the predicted tumor location. The DenseNet component started out with an initial convolution of 24 filters using a kernel size of seven with stride two and padding equal to three, followed by batch normalization (BN) [50], a ReLU activation function, and a max pooling layer with kernel size three and stride two, responsible for an initial spatial downsampling of the input by a factor of four. Three DenseNet blocks, comprised of four, eight, and 16 DenseNet layers with a transition block between each DenseNet block completed the processing of the latent representations. Each DenseNet layer was constructed by applying a growth rate of 16 and a bottleneck size of four. As with the UNet features, convolutional layers with 32 filters and kernel size one were applied to the output feature maps of each block. Different from the above description, BN was applied before the convolutional layer and ReLU afterwards. In configurations using the DenseNet branch, the obtained feature maps were again averaged over the spatial dimensions and flattened, yielding 96 additional features for outcome prediction, which were concatenated with the 128 UNet features for a total feature vector of dimension 224. Configurations without the DenseNet component only used the 128 features obtained from the UNet.

The resulting feature vector was then processed in parallel by up to two outcome heads in the multi-outcome setting (or a single one in single-outcome configurations). Each head consisted of layer normalization, followed by a fully connected output layer. Using a tanh activation function, the first outcome head produced an estimate of the log-hazard $\gamma \left(x\right)\in \left(-1,\;1\right)$ of the CPH model, which parameterizes the hazard rate *h* through

(1) $$h\left(t,x\right)=h_{0}\left(t\right)\cdot \exp\left(\gamma (x)\right).$$

Outputs of this head were optimized through minimization of the negative Cox partial log-likelihood function

(2) $$L_{\mathrm{Cox}}=-\sum_{i=1}^{n}\delta_{i}\left(\gamma \left(x_{i}\right)-\ln\left(\sum_{\begin{array}{c} j=1\\ t_{j}\geq t_{i} \end{array}}^{n}\exp\left(\gamma \left(x_{j}\right)\right)\right)\right),$$

where *n* denotes the batch size, δ∈{0,1} the binary event indicators with value one if and only if an event was observed, and *t* the corresponding event or censoring times.

The second outcome head was comprised of 10 numbers for producing predictions of the model suggested by Gensheimer et al. [41]. Through the application of a sigmoid activation function, the model outputs were the conditional survival probabilities for the 10 time intervals specified by the boundaries at [0, 6, 12, 18, 24, 30, 36, 48, 60, 84, 120] months. Unconditional survival probabilities for each (positive) timepoint were then obtained via cumulative products. We denote the corresponding negative log-likelihood loss function with $L_{\mathrm{GH}}$.

In the multi-outcome setting, the loss functions of both heads were added together to obtain

(3) $$L_{\mathrm{outcome}}=L_{\mathrm{Cox}}+L_{\mathrm{GH}},$$

while in single-outcome settings, only one of the terms was used. A segmentation loss $L_{\mathrm{seg}}=0.5\cdot \left(L_{\mathrm{Dice}}+L_{\mathrm{BCE}}\right)$, defined as the average between a Dice and binary cross-entropy (BCE) loss, was optionally added to the outcome loss $L_{\mathrm{outcome}}$ as an auxiliary task to steer feature learning towards tumor regions, forming the loss function $L_{\mathrm{outcome}}+L_{\mathrm{seg}}$. This yielded a total of four configurations (with and without segmentation loss and DenseNet branch) to evaluate for multi- and single-outcome settings.

**Figure 2:**
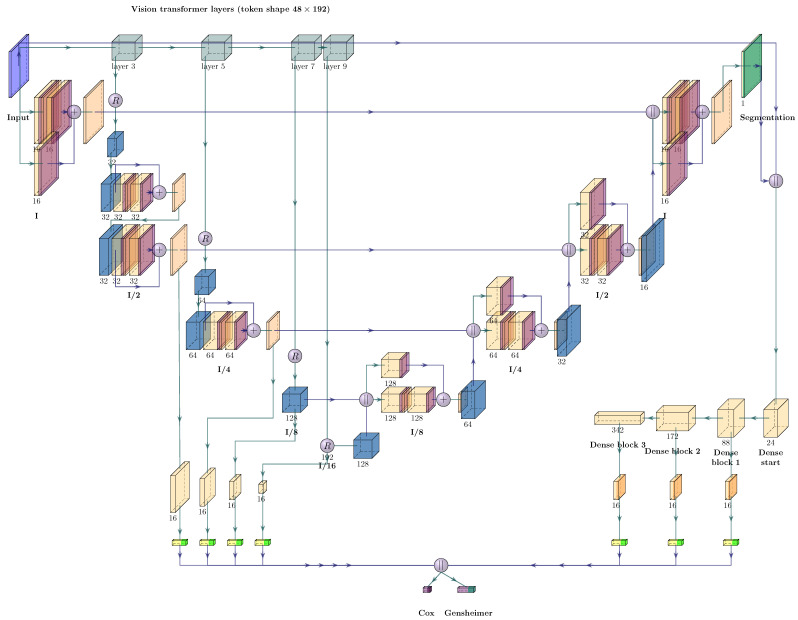
Architecture of the modified UNETR used for simultaneous outcome prediction and segmentation. A sigmoid activation (dark green) is applied to the output of the UNETR decoder to obtain segmentation predictions. For outcome prediction, convolutional layers (orange) with 16 filters of kernel size one are applied to the resulting feature maps of each encoder block, followed by a global average pooling (yellow), which reduces the spatial dimensions to a single number, before being flattened (light green) into 16-dimensional vectors and concatenated. Within each encoder block, vision transformer features of intermediate layers and the output layer are first reshaped (R symbol) and upsampled (blue) before being processed by residual blocks that contain convolutional layers with Leaky ReLU (α=0.01) activation. The decoder architecture resembles the one from a UNet and upsamples the incoming feature maps to twice their size, incorporating the corresponding encoder feature maps at the same time through concatenation.

Our ViT architecture for simultaneous segmentation and outcome prediction was based on the UNETR design proposed for segmentation tasks by Hatamizadeh et al. [51]. It extends the UNet shape to work with ViT models as feature extractors. Compared to the reference implementation of the used ‘MONAI’ library (https://monai.io/ (accessed on 4 October 2023), version 0.8.1), we reduced the number of model parameters by using a ViT with 9 transformer layers instead of 12, with six self-attention heads and a dimensionality of 64 per head. We also reduced the latent dimensionality to 192 and used an MLP dimension of 768. The extension of this architecture to perform outcome prediction was achieved analogously to the case described for the UNet and is depicted in Figure 2. For outcome prediction, features were derived from all four downsampling blocks and optionally from the DenseNet component. Here, we used 16 features of each downsampling and DenseNet block, for a total dimensionality of 112 and 64 when the DenseNet was present and absent, respectively.

### 2.5. Neural Network Training

During network training, the input images were initially cropped to a 1.25 times larger size of 60×80×80 around the tumor’s center of mass. Data augmentations were then performed by randomly choosing a crop of size 48×64×64 from the enlarged input region. Subsequently, the following augmentation operations were applied with a probability of 0.5 in each batch: Random image translations of a maximum of 25 voxels along each spatial dimension, addition of zero-mean Gaussian noise (standard deviation 0.1), random intensity shifts in the range of $\left[-2,\;2\right]$, random contrast adjustments ($\gamma \in \left[0.5,\;2.5\right]$), random Gaussian smoothing with variance in the range $\left[0.25,\;0.75\right]$ for each spatial dimension, random rotations of up to 20 degrees, and random axis flipping. All augmentation implementations were based on the ‘MONAI’ library (version 0.8.1). The neural networks were built and trained using the ‘PyTorch’ library (https://pytorch.org/ (accessed on 4 October 2023), version 1.11.0) for the Python programming language (version 3.8.10). Training of each neural network was carried out for 400 epochs using a batch size of 16. AdamW with a learning rate of $10^{-4}$ and a weight decay parameter of 0.1 was used as the optimizer. Weighted batch sampling was performed on the event status to approximately balance the number of events and censored samples contained in each batch, as suggested by Meng et al. [20]. During inference, the model weights that achieved the lowest loss on the internal test data during training were restored. As in the training step, enlarged image regions were initially extracted from the tumor’s center of mass. Then, model outputs were averaged from eight randomly selected crops without further augmentations. Our code is available from https://github.com/oncoray/multitask-hnscc (accessed on 4 October 2023).

### 2.6. Performance Evaluation

The predictive performance of the trained CV and ensemble models was analyzed separately for both the CPH and the Gensheimer head in the multi-outcome setting. Despite their joint training and sharing of the same input features, both heads were treated as independent models. For the Gensheimer head, evaluations were restricted to the predictions made at 24 months. The discriminative ability was measured using the concordance index (C-index) [52,53,54]. In a range between zero and one, it provides the degree of alignment between model predictions and observed event times between pairs of patients. For models that predict hazards (such as the CPH head), C-indices close to zero indicate optimal performance, as patients with shorter event times should be identified by receiving larger predicted hazards. On the other hand, for models producing probabilities of staying event-free (such as the Gensheimer head), values closer to one are preferable. Values around 0.5 indicate random predictions in both cases. For model ensembles, 95% confidence intervals of the C-indices are reported using the R package ‘survcomp’ [55,56]. As a second evaluation metric, we assessed whether model predictions allowed us to identify patient subgroups showing differing risks of event occurrence. Using the median predicted value of all patients used for training a model as a threshold, patients were assigned to two groups of low and high risk of an event. KM curves for the survival function for the analyzed endpoint were estimated for both groups and compared for differences using a log-rank test [57]. *P*-values below 0.05 were considered statistically significant.

## 3. Results

### 3.1. Multicentric DKTK CT Dataset

We evaluated single- and multi-outcome multitask models for the prediction of LRC and PFS on the DKTK and HECKTOR2021 datasets using four configurations of CNN and ViT models, trained with and without auxiliary segmentation loss and an additional DenseNet branch, respectively.

**Figure 3:**
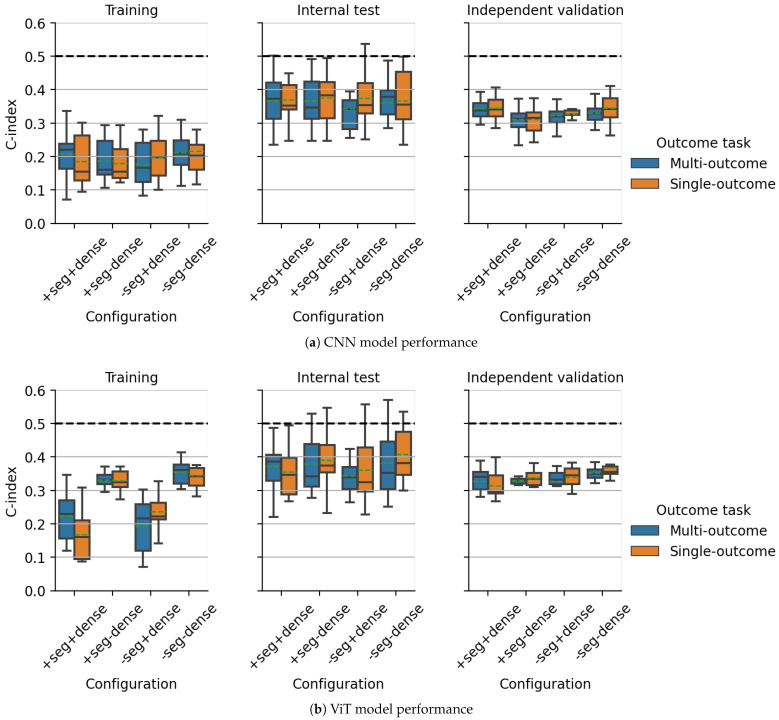
CPH head: cross-validation performance comparison between all investigated configurations of multi- and single-outcome models as measured by the concordance index. Model predictions are shown based on the 15 models trained during cross-validation for the endpoint loco-regional tumor control on the DKTK dataset using pre-treatment CT imaging data. ‘seg’ refers to incorporating an auxiliary segmentation loss, while ‘dense’ indicates the usage of an additional DenseNet branch. + and − signs denote presence and absence of a part of the architecture, respectively. The dashed horizontal line indicates a C-index of 0.5, which would be achieved by models making random predictions. Green dashed lines denote distribution means.

**Figure 4:**
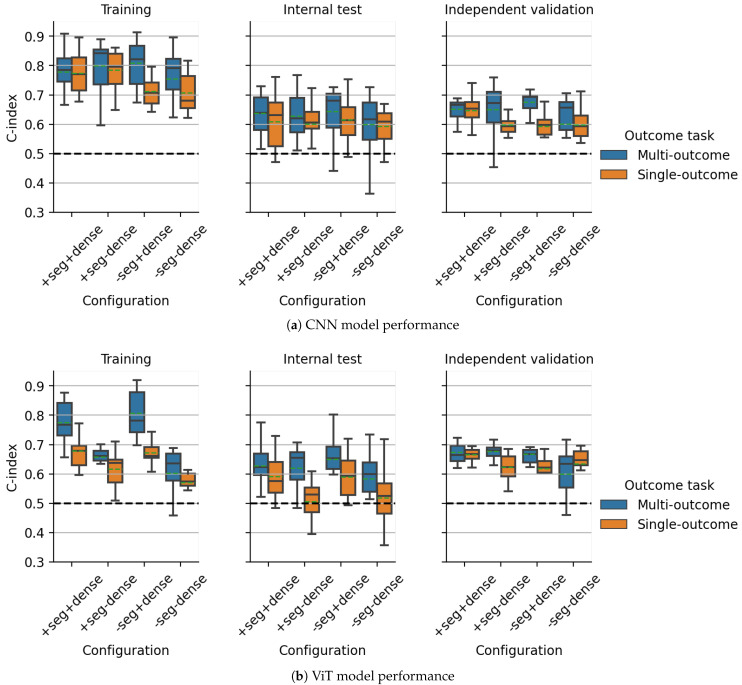
Gensheimer head (24 months): cross-validation performance comparison between all investigated configurations of multi- and single-outcome models as measured by the concordance index. Model predictions are shown based on the 15 models trained during cross-validation for the endpoint loco-regional tumor control on the DKTK dataset using pre-treatment CT imaging data. ‘seg’ refers to incorporating an auxiliary segmentation loss, while ‘dense’ indicates the usage of an additional DenseNet branch. + and − signs denote presence and absence of a part of the architecture, respectively. The dashed horizontal line indicates a C-index of 0.5, which would be achieved by models making random predictions. Green dashed lines denote distribution means.

For all experimental configurations evaluated on the DKTK dataset, the CV results for single- and multi-outcome models based on CNNs and ViTs are visualized in Figure 3 and Figure 4 for the predicted log-hazard of the CPH head and the predicted value of the survival function at 24 months predicted by the Gensheimer head, respectively. Independent validation performance based on the predicted hazard of the CPH head was similar for most investigated configurations and between single- and multi-outcome models for CNNs and ViTs, with average C-indices between 0.31 and 0.37. Discrimination on the internal test folds was slightly worse, with average C-indices between 0.34 and 0.41, and showed more variation between single- and multi-outcome models. The best CV performance for the CPH head was obtained by the single- and multi-outcome CNN without a DenseNet branch, trained with auxiliary segmentation loss (average C-index: 0.31, Figure 3a, right). Based on the value of the estimated survival function of the Gensheimer head at 24 months, the best average CNN model performance on the independent validation cohort was obtained by the multi-outcome CNN model with an additional DenseNet branch, trained without auxiliary segmentation loss (average C-index: 0.68, Figure 4a, right). Notably, multi-outcome models showed increased performance compared to single-outcome models across most configurations for CNNs and ViTs.

**Figure 5:**
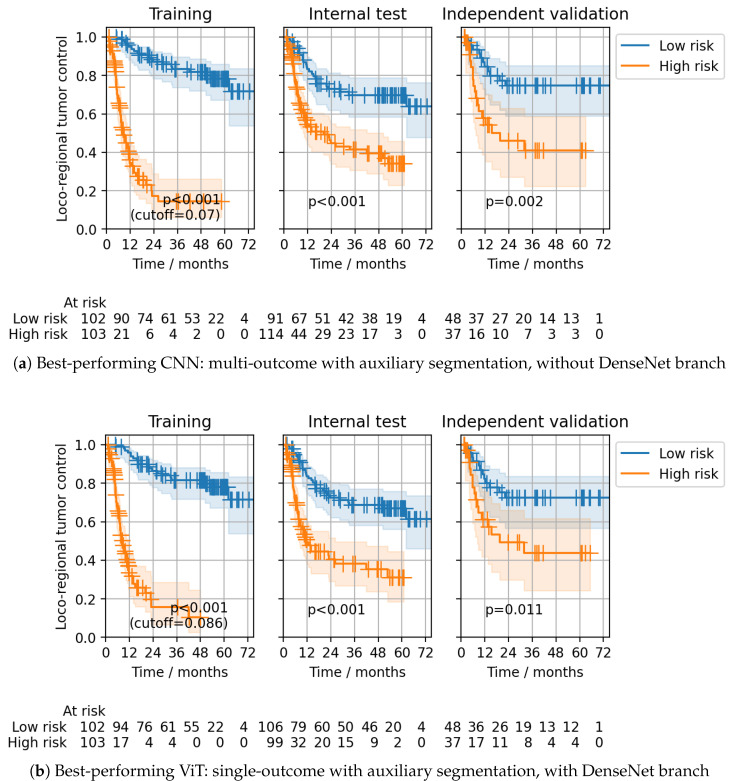
CPH head: ensemble stratifications obtained for the best-performing CNN and ViT configurations for the training, internal test, and independent validation data of the DKTK cohort. Stratifications into low (blue) and high (orange) risk groups of LRC were obtained by using the median prediction on the training data as a cutoff. *p*-values of the log-rank test for differences between the strata’s Kaplan–Meier curves are also provided. Transparently colored regions indicate 95% confidence intervals for the estimated survival functions.

**Figure 6:**
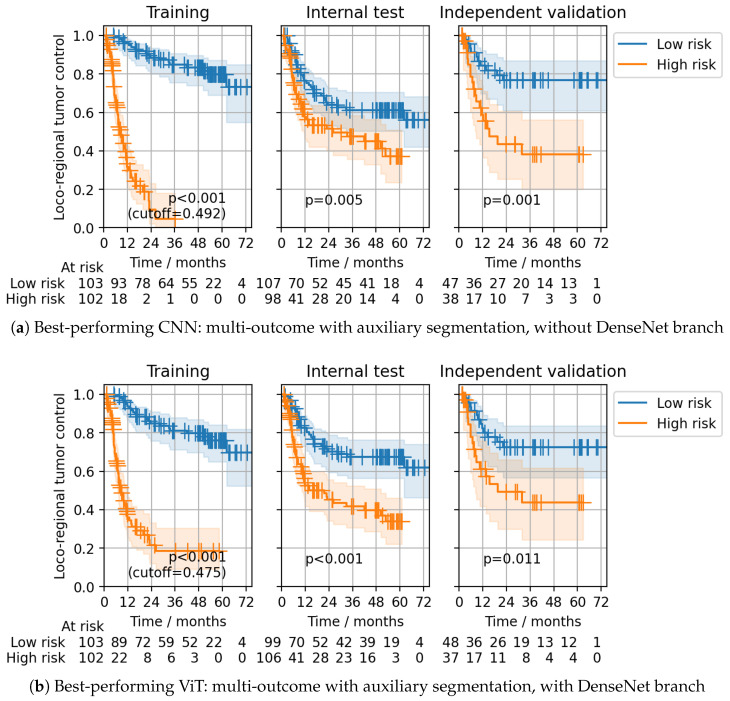
Gensheimer head (24 months): ensemble stratifications obtained for the best-performing CNN and ViT configurations for the training, internal test, and independent validation data of the DKTK cohort. Stratifications into low (blue) and high (orange) risk groups of LRC were obtained by using the median prediction on the training data as a cutoff. *p*-values of the log-rank test for differences between the strata’s Kaplan–Meier curves are also provided. Transparently colored regions indicate 95% confidence intervals for the estimated survival functions.

Model ensembling slightly improved the CPH model’s performance compared to the results obtained during CV. In line with the CV results, the best ensemble discrimination for the CPH head on the independent validation cohort was achieved using multi-outcome CNNs without the DenseNet branch, trained with auxiliary segmentation loss (C-index: 0.26 (0.18–0.34)) and single-outcome ViTs with the DenseNet branch, trained with auxiliary segmentation loss (C-index: 0.26 (0.18–0.35)) (Table 1). For both ensembles, statistically significant patient stratifications on the internal test and independent validation data were obtained (log-rank p=0.002 and 0.011, respectively) (Figure 5). The same multi-outcome CNN configuration that showed the best performance for the CPH head also achieved the best discrimination for the Gensheimer head at 24 months in independent validation (C-index: 0.74 (0.65–0.82)), as well as successful patient stratification (log-rank p=0.001, Figure 6a). The corresponding single-outcome model ensemble performed considerably worse (C-index: 0.62 (0.52–0.72)). Multi-outcome ViTs with the DenseNet branch and with auxiliary segmentation loss also showed good discrimination (C-index: 0.72 (0.62–0.81)) together with significant stratifications (log-rank p=0.011, Figure 6b). For ViTs, the gap between single- and multi-outcome performance was less pronounced.

### 3.2. Multicentric HECKTOR2021 PET/CT Dataset

CV results for all experimental configurations for the HECKTOR2021 PET/CT dataset are visualized in Supplementary Figures S2 and S3 for the CPH and Gensheimer heads, respectively.

The best internal test performance for the CPH head was achieved by multi-outcome CNNs and single-outcome ViTs without the DenseNet branch and trained with auxiliary segmentation loss with an average C-index of 0.33. In line with this, the same multi-outcome CNN showed the best discrimination during CV for the Gensheimer head with clear advantages compared to its corresponding single-outcome model (average C-index: 0.67 vs. 0.58) (Supplementary Figure S3a). Single- and multi-outcome ViTs with DenseNet and trained without auxiliary segmentation loss also achieved this level of performance (Supplementary Figure S3b).

The performance of the CNN and ViT model ensembles is provided in Supplementary Table S4. The best internal test discrimination for the CPH head was observed for ensembles of multi-outcome CNNs without DenseNet and trained with auxiliary segmentation loss (C-index: 0.29 (0.22–0.36)) which also showed the best performance on the DKTK data. Patient stratification into low- and high-risk groups of disease progression was also achieved (log-rank p=0.003, Supplementary Figure S4a). ViT ensembles performed slightly worse and showed the best discrimination and successful stratification for multi-outcome models with the DenseNet branch trained without auxiliary segmentation loss (C-index: 0.31 (0.24–0.39), log-rank p=0.002, Supplementary Figure S4b). Multi-outcome CNNs without the DenseNet branch and trained with auxiliary segmentation loss were found to show the best ensemble discrimination for the Gensheimer head as well (C-index: 0.71 (0.65–0.78)) and allowed for successful patient stratification (log-rank p=0.001, Supplementary Figure S5a). The best ViT performance was observed for multi-outcome ensembles trained with DenseNet branch and without auxiliary segmentation loss (C-index: 0.69 (0.62–0.76), log-rank p<0.001, Supplementary Figure S5b).

## 4. Discussion

We investigated how multitask training paradigms can affect the performance of outcome models for the prediction of PFS and LRC on two distinct multicentric datasets of patients with HNSCC, based on pre-treatment FDG-PET/CT and CT imaging, respectively. In addition, we evaluated and compared the performance of CNNs to models that combine convolutional layers and self-attention-based ViTs. We demonstrated the benefits of using a multitask learning paradigm for training DL-based predictors, implemented either through the incorporation of an auxiliary segmentation loss or through the usage of multi-outcome modeling, where two established complementary outcome models are jointly optimized. While the CPH model [35] can be used for the estimation of individual patient’s hazard risks independent of time, the model proposed by Gensheimer et al. [41] is able to provide estimates of the survival function at user-defined timepoints. We found ensembles of multi-outcome CNNs trained with auxiliary segmentation loss and without DenseNet branch to show the best performance consistently across both datasets and both outcome models, indicating their potential generalizability to other patient cohorts and treatment endpoints.

We analyzed the DKTK cohort in a previous study and derived a baseline model comprised of tumor volume as the only statistically significant clinical feature, with a C-index of 0.39. Morever, Leger et al. [9] evaluated traditional radiomics models on the same cohort and achieved an average independent validation C-index of 0.62 (corresponding to 0.38 in our context by inverting predictions). Compared to this, a 3D CNN based on the architecture of Hosny et al. [58] was found to perform better in predicting LRC using the CPH head [12]. This model consisted of a stack of four convolutional layers, comprised a total of 11.3 million parameters, and achieved a C-index of 0.31 (0.22–0.39) on the independent validation cohort in an ensemble of 30 models. In the current investigation, we achieved comparable or slightly improved CNN performance in all configurations using half the ensemble size. Moreover, our proposed models contained at most 1.48 million parameters when the DenseNet branch was included, while the best-performing multi-outcome CNN without the DenseNet branch consisted only of 0.37 million parameters. This confirms the attractiveness of the UNet architecture in application domains different from segmentation, and especially its effectiveness for outcome prediction tasks, as already pointed out by Baek et al. [21]. Moreover, as all the model architectures investigated in this work used volumetric data as input, this strengthens and supports our previous findings that 3D models trained from scratch can offer adequate predictive performance and should be the first option when working on outcome prediction problems based on CT imaging data.

The HECKTOR2021 dataset was previously analyzed using multitask ViT models for the simultaneous segmentation and prediction of PFS by Saeed et al. [59]. In contrast to our investigation, they optimized the loss function proposed by Yu et al. [25] for outcome modeling and did not investigate the impact of having more than one outcome model. Moreover, while the focus of our current work was to derive predictive models exclusively based on imaging data, they combined imaging input with tabular clinical features and obtained an average five-fold CV C-index of 0.76 using a single repetition, which was better than our best average single-outcome-ViT performance for the CPH output, with a C-index of 0.33 (corresponding to a C-index of 0.67 if predictions were negated), albeit at an increased standard deviation of 0.14 compared to ours of 0.10, which might be due to our three repetitions. Therefore, the integration of clinical parameters into the investigated architectures provides an interesting direction for future work and is likely to improve performance. Meng et al. [14] evaluated their ‘DeepMTS’ architecture, that we derived our CNN models from, on the HECKTOR 2022 challenge data, a superset of more than twice the size of the data available to us. They also used the CPH head for outcome predictions and achieved an average five-fold CV C-index (one repetition) of 0.71 when only using imaging data. Additionally, including radiomics features obtained from the predicted segmentation masks and incorporating them with the predicted risk score and clinical information in a CPH model, they improved this score to 0.77, indicating further room for improving the prediction quality through complementary data sources.

Interestingly, we observed slightly improved model performance on the DKTK data compared to the HECKTOR2021 challenge data, even though both were of comparable size and the latter provided additional metabolic information coming from the PET imaging data, which were not present in the DKTK cohort. This observation could be attributable to inherent patient differences between both cohorts. The DKTK cohort was comprised predominantly of patients presenting with disease T stage 3 or 4 (more than 80% of cases), while more than half of the patients of the HECKTOR2021 cohort were assigned to T stage 1 or 2. Also, among patients for which information about infection with HPV was available, less than 15% were HPV positive in the DKTK cohort, while this was the case for about 75% of patients in the HECKTOR2021 cohort. Moreover, median tumor volumes were smaller in the HECKTOR2021 cohort, which might complicate prognosis. Additionally, the different treatment endpoints or the smaller number of models used for ensembling could also have had an effect. As an independent validation cohort was available for the DKTK data, ensemble averaging was performed across 15 models, while for each patient of the HECKTOR2021 dataset, the ensemble prediction of each patient considered only three model predictions (coming from the models trained in the folds that the patient was part of the internal test data).

While we did not find strong evidence for supporting our initial hypothesis, that the inclusion of the Gensheimer model might lead to performance improvements of the CPH head due to enhanced loss feedback during training, the performance of the Gensheimer model was noticeably improved for most CNN- and ViT-based multi-outcome configurations on the DKTK data and the best-performing configuration on the HECKTOR2021 dataset.

Leveraging multitask learning is an important step towards the clinical use of models as decision support tools. We found that multitask models (i) performed better than clinical or traditional radiomics models. Moreover, they achieve similar or better performance than our previously evaluated CNN models despite only using about 10% of their parameters, thereby improving computational and resource utilization. (ii) In addition, multitask models trained with an auxiliary segmentation objective only require a rough indication of the tumor location. Thus, they require less manual contouring prior to application on patient imaging data than traditional radiomics approaches.

In future investigations, the multitask approach could be further extended through the introduction of additional auxiliary objectives, such as input reproduction through an autoencoder [28], the auxiliary prediction of handcrafted radiomics features [60] known to be prognostic for outcome, classification tasks, or the estimation of clinical patient attributes to guide learning of potentially relevant and humanly interpretable features. Similarly, further outcome heads could be introduced. We initially investigated the inclusion of two additional models into the multi-outcome setup: a fully parametric log-normal model [61,62,63] and one that directly optimized an approximation of the C-index metric [54]. Unfortunately, including either of these, we encountered numerical problems when training the neural networks, as undefined values were repeatedly produced. We, therefore, refrained from further exploring these options. This demonstrates that proper handling of multitask approaches can become difficult if too many tasks are incorporated that could interact and compete with each other in complex ways during model optimization. To further illustrate this, we found that while outcome predictive performance for multitask models was not negatively affected by the introduction of a DenseNet branch in most configurations, segmentation performance was noticeably reduced by its incorporation on both datasets, as shown in the boxplots of Supplementary Figure S6. The reason for this observation could likely be that the DenseNet branch, introduced to support the outcome prediction, led to a shift in focus towards the outcome prediction task and reduced segmentation performance. This was especially apparent for CNNs, as the DenseNet contained about three times the number of parameters compared to the plain UNet, a limitation of our initial architecture design. On the same note, we observed slightly improved segmentation performance for ViTs compared to CNNs in both cohorts, likely due to the much larger network size, but possibly also attributable to the incorporated self-attention mechanism, designed to allow for information exchange between non-local image neighborhoods and a better focus on smaller image details. These observations suggest that the main modeling objectives need to be clearly defined when planning multitask experiments, as it is unlikely that a model trained using a multitask paradigm will do equally well on all tasks. For our experiments, however, a drop in segmentation performance was not detrimental, as the focus was on outcome modeling and the segmentation objective was merely introduced as an auxiliary task to guide model training towards image regions containing tumor tissue. In general, different weighting strategies for the individual parts of the multitask loss function could be explored in future work, which might reduce such problems. Moreover, it might be worth exploring transitioning from multitask learning, where different objectives are scalarized into a single objective [64], to full-scale multi-objective optimization approaches that attempt to find Pareto optimal solutions for a vector of objectives [65,66].

Further limitations of our work include the relatively small number of patients included for model training, which led to apparent signs of overfitting, as the predictions on patients in the training cohorts showed much better performance compared to those patients not used for model training. In line with this are the relatively large fluctuations in our performance metrics during CV as well as wide confidence intervals for ensemble metrics. Moreover, our models were trained using imaging data from only a limited number of hospitals in Europe and Canada. We, therefore, do not expect our trained models to be directly applicable to patients from other geographic areas with non-comparable outcome characteristics or those treated at sites with diverging imaging protocols. Nevertheless, based on its good performance on both evaluated cohorts, we are confident that our identified CNN architecture trained in a multi-outcome setup with auxiliary segmentation loss and without a DenseNet branch can achieve promising performance when trained on data from different centers and may, thus, form a sensible baseline model in future analyses. Another limitation comes from the fact that we analyzed the predictive performance of the Gensheimer head only at a single timepoint for the sake of comparability to the CPH head, even though predictions for multiple timepoints were provided. For an evaluation of the predictive quality at all timepoints and their calibration, other metrics, such as the integrated Brier score, could be more appropriate [67]. Related to this, we currently did not closely investigate the degree of concordance between the predictions coming from both outcome heads for the trained multi-outcome models. Ways of achieving coupling between predictions from the different outcome heads to ensure, e.g., that high hazards predicted by the CPH head are in line with low values for the survival function obtained by the Gensheimer model, might also indicate a direction towards increasing model performance.

CNN and ViT models in our experiments differed noticeably in the number of parameters, which may limit a direct comparison of the two approaches for outcome modeling. The dramatically larger size of our ViT models compared to the CNNs was due to the fact that, in preliminary experiments, smaller ViT models could not be successfully trained to minimize the loss function. This adds to the existing evidence that, owing to their lack of inductive biases such as translation invariance, ViT models might only show their full potential in large scale models and when trained on sufficiently large datasets.

Apart from further extending the multitask paradigm, a different avenue towards improving the predictive performance of DL algorithms lies in the application of self-supervised pre-training strategies for CNNs [68,69,70] and ViTs [71,72,73,74]. In such approaches, models are pre-trained on auxiliary tasks that do not require labeled data, aiming to establish suitable feature representations that can be leveraged in a second step through fine-tuning on the target task using the actual label information. Adoption of these pre-training strategies might offer fruitful directions for successful training of outcome prediction models, especially in situations of limited dataset sizes, that frequently occur when working on medical imaging data, as presented in this work.

## 5. Conclusions

Multitask learning configurations for CNN and ViT models, implemented either through the incorporation of two outcome heads, an auxiliary segmentation objective, or a combination of both, showed strong prognostic performance for the endpoints loco-regional tumor control and progression-free survival on two distinct multicentric datasets of CT and PET/CT imaging data, respectively. Multi-outcome CNNs trained with an auxiliary segmentation loss were identified as the best-performing models for both outcome objectives and datasets. We plan to validate the proposed models in a prospective multicentric cohort that recently completed recruitment to assess their generalizability and facilitate their clinical translation.

## Figures and Tables

**Table 1:** Discriminative performance as measured by the concordance index for model ensembles for the prediction of loco-regional tumor control on the DKTK dataset using multi-outcome CNN and ViT models. A * identifies statistically significant stratifications of the ensemble model on the internal test and independent validation data (log-rank p<0.05). Values in parentheses denote 95% confidence intervals as computed by the ‘concordance.index’ function from the R package ‘survcomp’. Best independent validation performances for both outcome heads are marked in bold, separately for CNNs and ViTs.

Configuration		Head	CNN			ViT		
**Seg**	**Dense**		**Training**	**IT**	**IV**	**Training**	**IT**	**IV**
**Multi-Outcome (CPH + GH)**								
✓	✓	CPH	0.15 (0.11–0.18)	0.35 * (0.29–0.40)	0.33 * (0.24–0.43)	0.15 (0.12–0.19)	0.36 * (0.30–0.42)	0.29 * (0.19–0.38)
		GH (24 m)	0.86 (0.83–0.89)	0.65 * (0.60–0.71)	0.67 (0.57–0.76)	0.87 (0.84–0.90)	0.65 * (0.59–0.71)	**0.72** * **(0.62–0.81)**
✓	✗	CPH	0.13 (0.10–0.16)	0.36 * (0.30–0.42)	**0.26** * **(0.18–0.34)**	0.32 (0.26–0.38)	0.38 * (0.32–0.44)	0.31 * (0.22–0.40)
		GH (24 m)	0.89 (0.86–0.92)	0.64 * (0.58–0.70)	**0.74** * **(0.65–0.82)**	0.68 (0.62–0.74)	0.60 * (0.54–0.67)	0.70 * (0.61–0.79)
✗	✓	CPH	0.11 (0.08–0.13)	0.33 * (0.27–0.39)	0.29 * (0.20–0.38)	0.12 (0.09–0.15)	0.32 * (0.27–0.38)	0.30 * (0.22–0.39)
		GH (24 m)	0.90 (0.88–0.92)	0.66 * (0.61–0.72)	0.72 * (0.63–0.80)	0.90 (0.87–0.92)	0.66 * (0.60–0.72)	0.71 * (0.62–0.79)
✗	✗	CPH	0.15 (0.12–0.19)	0.36 * (0.30–0.41)	0.30 * (0.22–0.39)	0.32 (0.26–0.38)	0.39 * (0.33–0.45)	0.32 * (0.23–0.41)
		GH (24 m)	0.88 (0.85–0.90)	0.62 * (0.56–0.68)	0.69 * (0.60–0.78)	0.68 (0.61–0.74)	0.60 (0.54–0.66)	0.68 * (0.59–0.77)
**Single-Outcome (CPH)**								
✓	✓	CPH	0.12 (0.09–0.14)	0.34 * (0.28–0.40)	0.32 * (0.23–0.41)	0.11 (0.09–0.14)	0.33 * (0.27–0.38)	**0.26** * **(0.18–0.35)**
✓	✗	CPH	0.13 (0.10–0.16)	0.34 * (0.28–0.40)	0.28 * (0.19–0.37)	0.31 (0.25–0.36)	0.42 (0.36–0.48)	0.32 * (0.23–0.41)
✗	✓	CPH	0.14 (0.11–0.18)	0.37 * (0.31–0.42)	0.32 * (0.23–0.40)	0.16 (0.12–0.19)	0.36 * (0.29–0.42)	0.33 (0.24–0.42)
✗	✗	CPH	0.15 (0.11–0.18)	0.36 * (0.30–0.42)	0.31 * (0.22–0.39)	0.33 (0.27–0.39)	0.45 * (0.38–0.51)	0.34 * (0.25–0.44)
**Single-Outcome (GH)**								
✓	✓	GH (24 m)	0.87 (0.84–0.90)	0.63 * (0.57–0.69)	0.68 * (0.59–0.77)	0.77 (0.72–0.82)	0.59 * (0.53–0.65)	0.69 * (0.60–0.79)
✓	✗	GH (24 m)	0.84 (0.81–0.88)	0.62 * (0.56–0.68)	0.62 * (0.52–0.72)	0.68 (0.62–0.74)	0.52 (0.46–0.59)	0.67 * (0.57–0.76)
✗	✓	GH (24 m)	0.77 (0.73–0.82)	0.62 * (0.56–0.68)	0.62 (0.53–0.72)	0.73 (0.68–0.79)	0.56 * (0.50–0.63)	0.65 (0.56–0.75)
✗	✗	GH (24 m)	0.80 (0.76–0.84)	0.59 (0.53–0.65)	0.64 (0.54–0.74)	0.61 (0.54–0.67)	0.54 (0.48–0.60)	0.69 * (0.59–0.78)

Abbreviations: CNN, convolutional neural network; CPH, Cox proportional hazards model; Dense, DenseNet branch; GH, Gensheimer model; IT, internal test; IV, independent validation; m, months; Seg, segmentation loss; ViT, vision transformer.

## Data Availability

The data presented in this study are available on reasonable request from the corresponding author. The data are not publicly available due to local regulations on the protection of patient data. Analysis code is available from https://github.com/oncoray/multitask-hnscc (accessed on 4 October 2023).

## Supplementary Materials

The following supporting information can be downloaded at: https://www.mdpi.com/article/10.3390/cancers15194897/s1, Figure S1: Kaplan–Meier curve for exploration and independent validation data of the DKTK cohort; Figure S2: CV performance for the CPH head on the HECKTOR2021 cohort; Figure S3: CV performance for the Gensheimer head on the HECKTOR2021 cohort; Figure S4: Kaplan–Meier plots for ensemble stratifications for the CPH head on the HECKTOR2021 cohort; Figure S5: Kaplan–Meier plots for ensemble stratifications for the Gensheimer head on the HECKTOR2021 cohort; Figure S6: CV performance of segmentation quality for the DKTK and HECKTOR2021 cohorts; Table S1: Patient characteristics of the DKTK cohort; Table S2: Image acquisition parameters of the DKTK cohort; Table S3: Patient characteristics of the HECKTOR2021 cohort; Table S4: Ensemble performances on the HECKTOR2021 cohort.
