# Supplementary material for: Multitask Learning with Convolutional Neural Networks and Vision Transformers Can Improve Outcome Prediction for Head and Neck Cancer Patients
Source: Cancers (Basel). 2023 Oct 9;15(19):4897. doi: 10.3390/cancers15194897 (PMC10571894; doi:10.3390/cancers15194897)
Supplement: Supplementary file 1 [file cancers-15-04897-s001.zip › cancers-2613389-supplementary.pdf]

Supplementary Materials: Multitask Learning with Convolutional Neural Networks and Vision Transformers Can Improve Outcome Prediction for Head and Neck Cancer Patients

Table S1. Patient characteristics of the exploratory and independent validation cohort of the DKTK dataset: *P*-values were obtained using two-sided Mann-Whitney U-tests for continuous variables and χ^2 homogeneity tests for categorical variables.

Variable	Exploratory cohort (n=205)		Independent validation cohort (n=85)		*p*-value
	Median	(range)	Median	(range)	
Follow up time of patients alive (months)	53	(4–132)	43	(8–107)	0.72
Observed loco-regional recurrence time (months)	8	(1–128)	9	(2–32)	0.99
Primary tumor volume (cm^3)	29.13	(4.52–321.74)	40.62	(2.70–239.07)	0.038
Age (years)	59.00	(39.20–84.50)	55.00	(37.00–76.00)	0.025
	Number of patients	(%)	Number of patients	(%)	
Observed loco-regional tumor recurrence	83	(40)	28	(33)	0.28
Gender					
male/female	173/32	(84/16)	74/11	(87/13)	0.69
cT-stage					
T1/T2/T3/T4	2/23/50/130	(1/11/25/63)	2/9/30/44	(2/11/35/52)	0.19
cN-stage					
N0/N1/N2/N3/unknown	30/7/153/15/0	(15/3/75/7/0)	9/8/64/3/1	(11/9/75/4/1)	0.069
UICC-stage					
I/II/III/IV	0/0/15/190	(0/0/7/93)	1/2/9/73	(1/2/11/86)	0.040
Tumor site					
oropharynx/oral cavity/hypopharynx/larynx	93/50/62/0	(45/25/30/0)	29/23/28/5	(34/27/33/6)	0.003
p16 status					
negative/positive/unknown	147/28/30	(72/13/15)	52/5/28	(61/6/33)	0.25
Pathological grading					
0/1/2/3/unknown	1/6/131/60/7	(1/3/64/29/3)	0/0/43/35/7	(0/0/51/41/8)	0.063
Smoking status					
no/yes/unknown	40/163/2	(20/79/1)	13/51/21	(15/60/25)	1.00
Alcohol consumption					
no/yes/unknown	62/85/58	(30/41/29)	23/25/37	(27/29/44)	0.60

Table S2. CT image acquisition parameters of the exploratory and independent validation cohort of the DKTK dataset.

Variable	Exploratory cohort (n=205)		Independent validation cohort (n=85)	
	Median	(range)	Median	(range)
Exposure time (ms)	1000	(500–1000)	500	(500–750)
Exposure (mAs)	146	(31–300)	61	(22–150)
Tube current (mA)	162	(32–440)	183	(68–438)
	Number of patients	(%)	Number of patients	(%)
Manufacturer				
Siemens/GE/Philips/unknown	72/31/20/82	35/15/10/40	66/14/0/5	78/16/0/6
Spacing z (mm)				
2/2.5/3/3.75/5	20/22/63/1/99	10/11/30/1/48	0/0/27/0/58	0/0/32/0/68
Spacing x/y (mm)				
<0.98/0.98/1.17/1.27/1.37	20/110/20/26/29	10/53/10/13/14	0/13/0/14/58	0/15/0/17/68
Kilovoltage peak (keV)				
120/130/140/unknown	97/2/18/88	47/1/9/43	66/0/0/19	78/0/0/22
Reconstruction kernel				
B	20	10	0	0
B10s	0	0	1	1
B20f	3	1	52	62
B20s	0	0	1	1
B30f	2	1	0	0
B30s	27	13	0	0
B31f	0	0	12	14
B31s	20	10	0	0
B40f	1	1	0	0
B40s	0	0	0	0
STANDARD	23	11	0	0
59.10.AB50	18	9	0	0
unknown	91	44	19	22

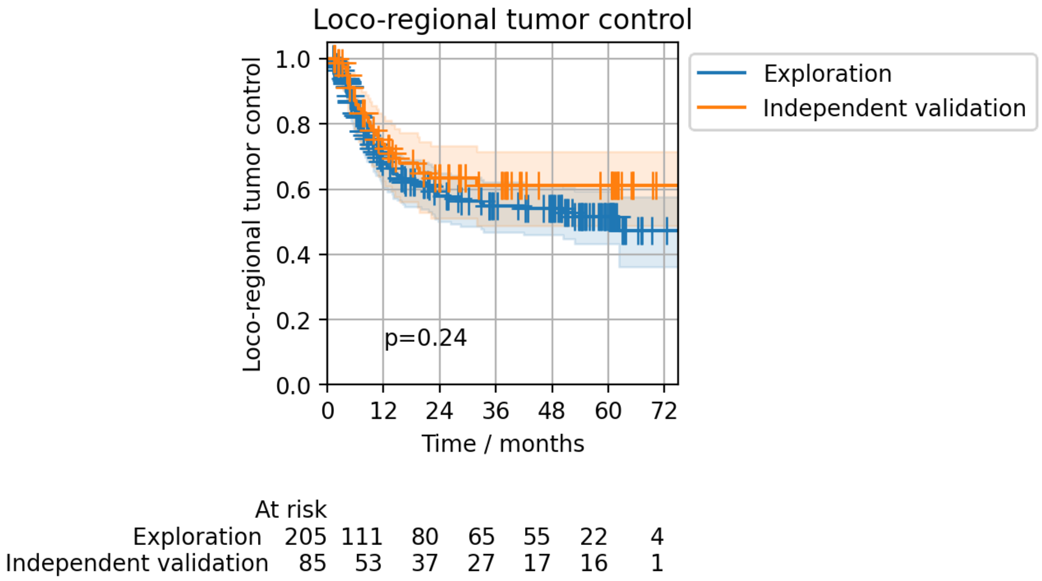

Figure S1. A comparison of the Kaplan-Meier estimates for the endpoint loco-regional tumor control between the exploration and independent validation cohort of the DKTK data. The *p*-value of the log-rank test for differences between both functions is reported as well.

Table S3. Patient characteristics of the HECKTOR2021 cohort.

Variable	HECKTOR2021 Cohort (n = 224)	
	**Median**	**(Range)**
Age (years)	63	(34–90)
Primary tumor volume (cm^3)	8.26	(0.57–186.44)
Follow-up time without progression (months)	43	(22–101)
Observed progression time (months)	16	(3–63)
	**Number of Patients**	**(%)**
Observed progression	56	(25)
Gender		
male/female	167 / 57	(75 / 25)
cT-stage		
T1/T2/T3/T4/unknown	26/94/58/45/1	(12/42/26/20/0)
cN-stage		
N0/N1/N2/N3	33/26/150/15	(15/12/66/7)
UICC-stage		
I/II/III/IV	4/19/29/172	(2/8/13/77)
HPV status		
negative/positive/unknown	30/84/110	(13/38/49)
Smoking status		
no/yes/unknown	5/18/201	(2/8/90)
Alcohol consumption		
no/yes/unknown	11/12/201	(5/5/90)
Chemotherapy		
no/yes	27/197	(12/88)

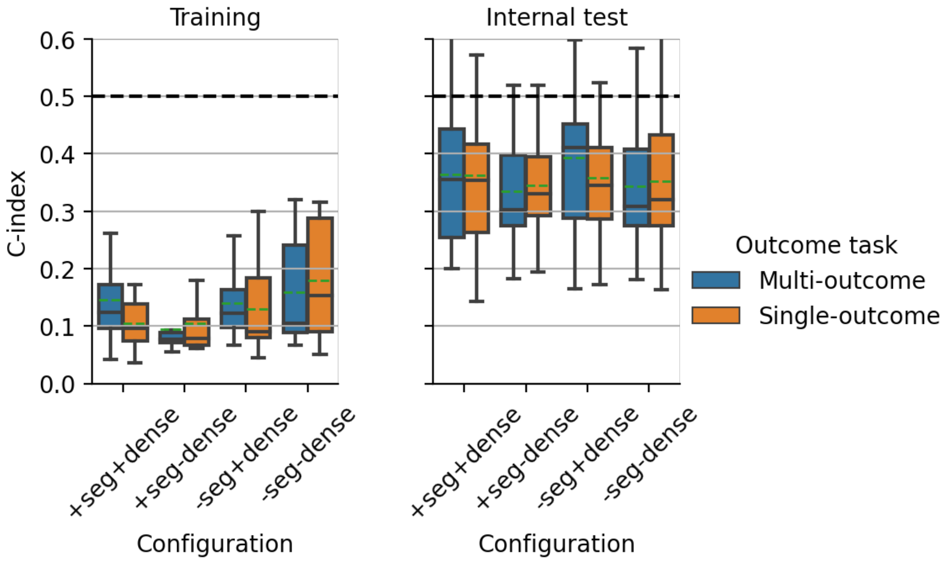

(a) CNN model performance

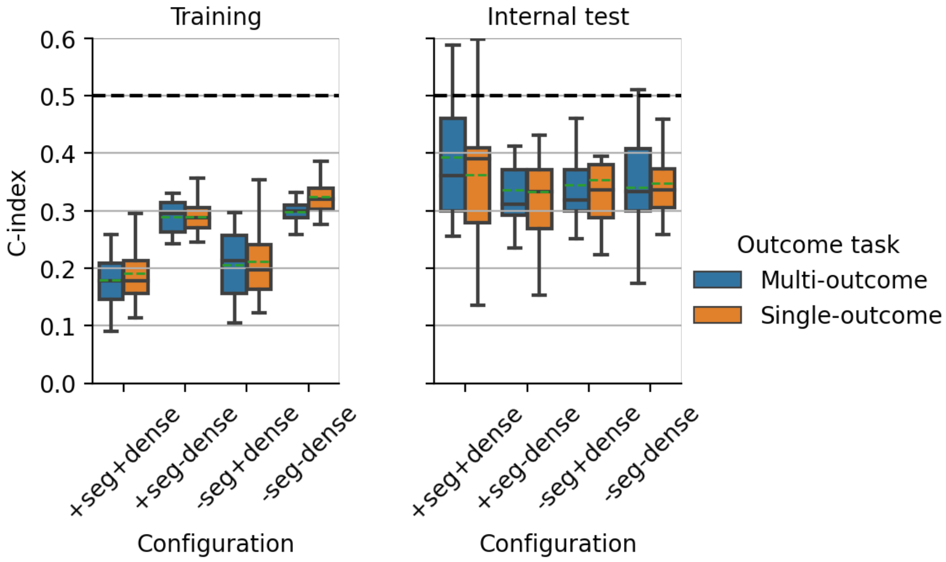

(b) ViT model performance

Figure S2. CPH head: cross-validation performance comparison between all investigated configurations of multi- and single-outcome models as measured by the concordance index. Model predictions are shown based on the 15 models trained during cross-validation for the endpoint progression-free survival on the HECKTOR2021 challenge training dataset using pre-treatment PET/CT imaging data. 'seg' refers to incorporating an auxiliary segmentation loss, while 'dense' indicates the usage of an additional DenseNet branch. $+$ and $-$ signs denote presence and absence of a part of the architecture, respectively. The dashed horizontal line indicates a C-index of 0.5, which would be achieved by models making random predictions. Green dashed lines denote distribution means.

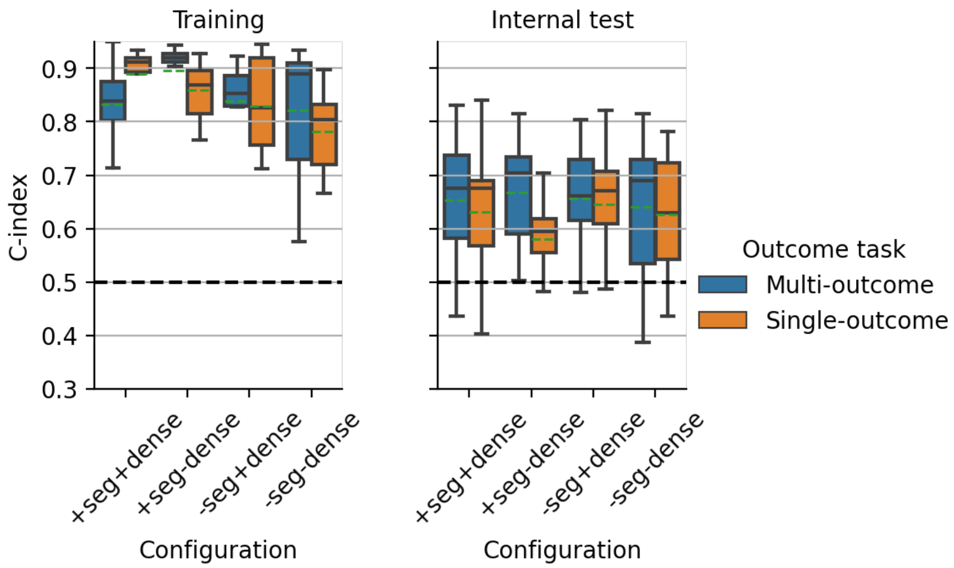

(a) CNN model performance

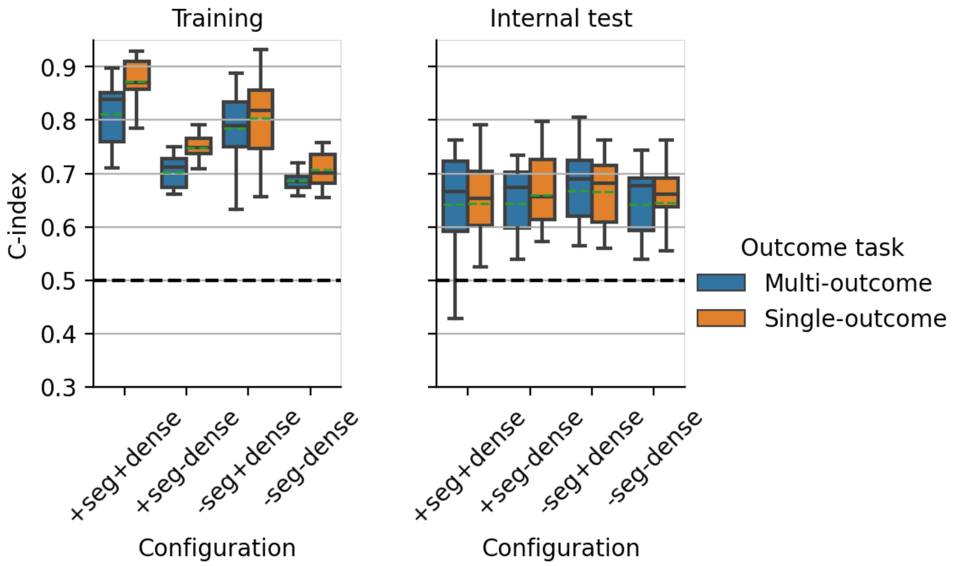

(b) ViT model performance

Figure S3. Gensheimer head (24 months): cross-validation performance comparison between all investigated configurations of multi- and single-outcome models as measured by the concordance index. Model predictions are shown based on the 15 models trained during cross-validation for the endpoint progression-free survival on the HECKTOR2021 challenge training dataset using pre-treatment PET/CT imaging data. 'seg' refers to incorporating an auxiliary segmentation loss, while 'dense' indicates the usage of an additional DenseNet branch. + and − signs denote presence and absence of a part of the architecture, respectively. The dashed horizontal line indicates a C-index of 0.5, which would be achieved by models making random predictions. Green dashed lines denote distribution means.

Table S4. Discriminative performance as measured by the concordance index for model ensembles for the prediction of progression-free survival on the HECKTOR2021 training dataset using multitask CNN and ViT models. A * identifies statistically significant stratifications of the ensemble model on the internal test data (log-rank $p < 0.05$). Values in parentheses denote 95% confidence intervals as computed by the 'concordance.index' function from the R package 'survcomp'. Best internal test performances for both outcome heads are marked in bold, separately for CNNs and ViTs.

Configuration		Head	CNN		ViT	
Seg	Dense		Training	IT	Training	IT
**Multi-outcome (CPH + GH)**						
✓	✓	CPH	0.07 (0.05–0.10)	0.33* (0.26–0.41)	0.12 (0.08–0.17)	0.35* (0.26–0.43)
		GH (24m)	0.90 (0.86–0.93)	0.69* (0.61–0.76)	0.86 (0.82–0.91)	0.68* (0.60–0.75)
✓	✗	CPH	0.06 (0.04–0.09)	**0.29*** **(0.22–0.36)**	0.26 (0.20–0.32)	0.37* (0.30–0.44)
		GH (24m)	0.94 (0.92–0.97)	**0.71*** **(0.65–0.78)**	0.72 (0.66–0.78)	0.64* (0.57–0.72)
✗	✓	CPH	0.08 (0.05–0.10)	0.32* (0.25–0.39)	0.13 (0.09–0.18)	**0.31*** **(0.24–0.39)**
		GH (24m)	0.90 (0.86–0.94)	0.67* (0.60–0.75)	0.86 (0.82–0.91)	**0.69*** **(0.62–0.76)**
✗	✗	CPH	0.07 (0.04–0.10)	0.31* (0.24–0.38)	0.28 (0.21–0.34)	0.41 (0.33–0.48)
		GH (24m)	0.94 (0.92–0.96)	0.66 (0.59–0.73)	0.70 (0.63–0.76)	0.64* (0.57–0.71)
**Single-outcome (CPH)**						
✓	✓	CPH	0.07 (0.05–0.10)	0.33* (0.25–0.41)	0.13 (0.09–0.17)	0.37* (0.29–0.45)
✓	✗	CPH	0.06 (0.04–0.08)	0.35* (0.27–0.42)	0.28 (0.21–0.34)	0.38 (0.31–0.45)
✗	✓	CPH	0.05 (0.04–0.07)	0.37 (0.29–0.45)	0.13 (0.08–0.17)	0.35* (0.27–0.44)
✗	✗	CPH	0.08 (0.05–0.11)	0.31* (0.24–0.37)	0.32 (0.26–0.38)	0.37 (0.30–0.44)
**Single-outcome (GH)**						
✓	✓	GH (24m)	0.92 (0.89–0.95)	0.68* (0.61–0.75)	0.91 (0.87–0.95)	0.67* (0.60–0.74)
✓	✗	GH (24m)	0.90 (0.86–0.94)	0.60 (0.52–0.67)	0.78 (0.72–0.83)	0.65* (0.57–0.72)
✗	✓	GH (24m)	0.91 (0.87–0.94)	0.67* (0.60–0.74)	0.88 (0.85–0.92)	0.68* (0.62–0.75)
✗	✗	GH (24m)	0.86 (0.81–0.91)	0.68* (0.61–0.75)	0.73 (0.67–0.79)	0.65* (0.58–0.72)

Abbreviations: CNN, convolutional neural network; CPH, Cox proportional hazards model; Dense, DenseNet branch; GH, Gensheimer model; IT, internal test; IV, independent validation; m, months; Seg, segmentation loss; ViT, vision transformer

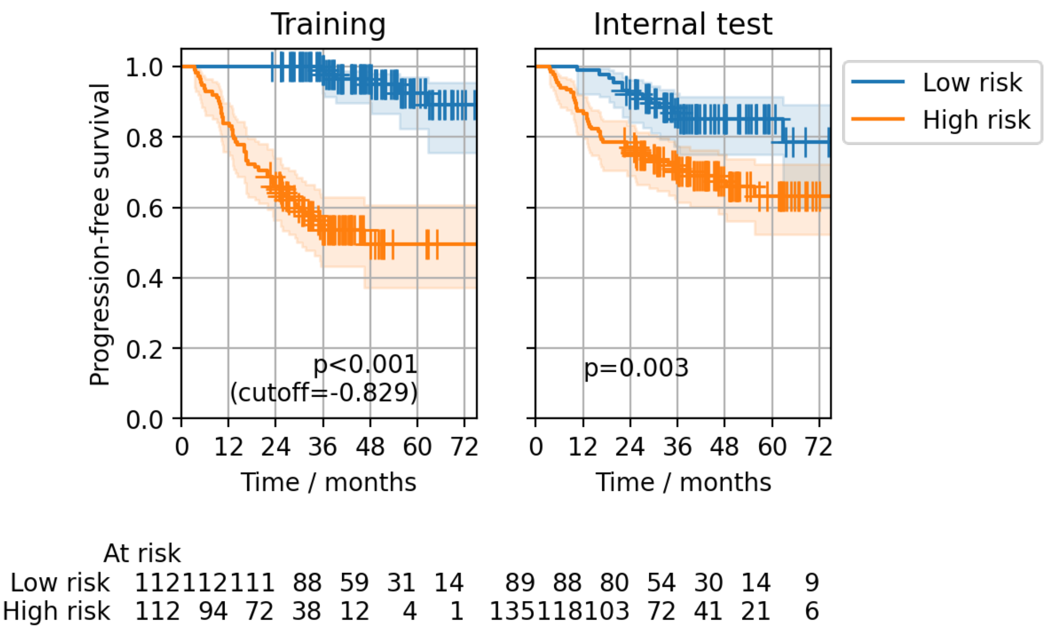

(a) Best-performing CNN: multi-outcome with auxiliary segmentation, without DenseNet branch

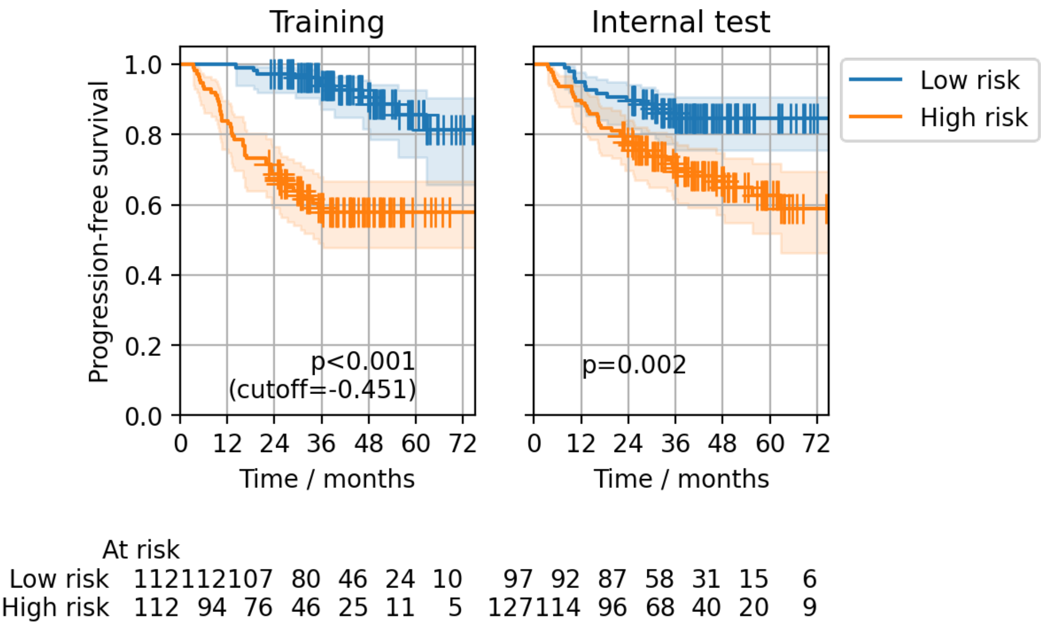

(b) Best-performing ViT: multi-outcome without auxiliary segmentation, with DenseNet branch

Figure S4. CPH head: ensemble stratifications obtained for the best-performing CNN and ViT configurations for the training and internal test data of the HECKTOR2021 cohort. Stratifications into low (blue) and high (orange) risk groups of LRC were obtained by using the median prediction on the training data as a cutoff. *P*-values of the log-rank test for differences between the strata's Kaplan-Meier curves are also provided. Transparently colored regions indicate 95% confidence intervals for the estimated survival functions.

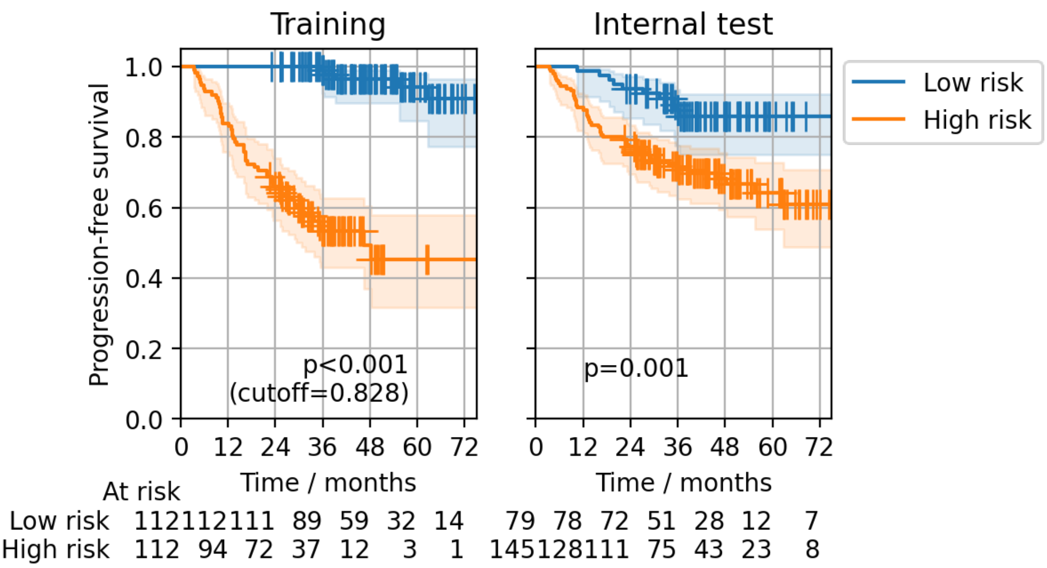

(a) Best-performing CNN: multi-outcome with auxiliary segmentation, without DenseNet branch

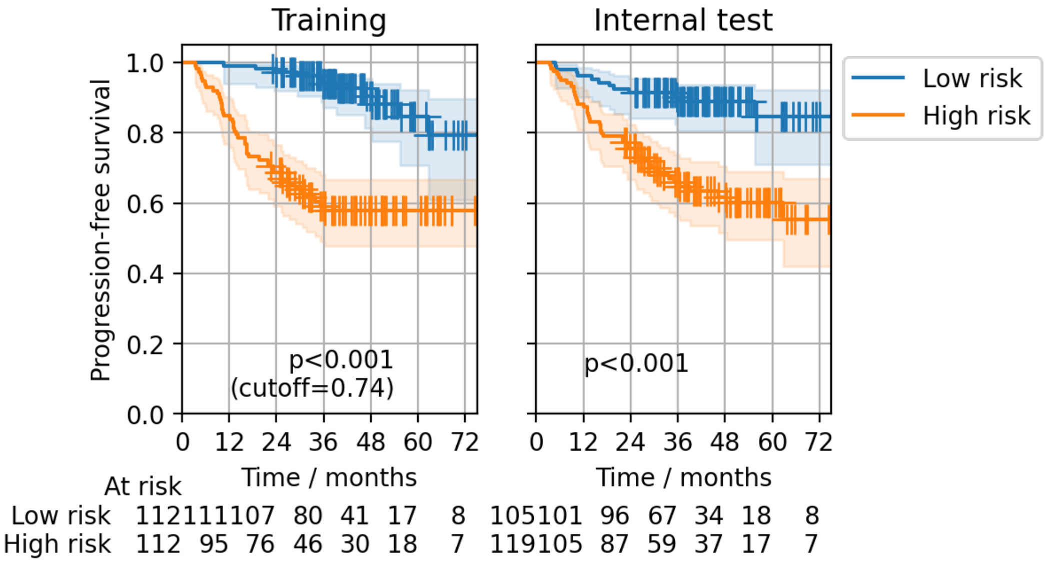

(b) Best-performing ViT: multi-outcome without auxiliary segmentation, with DenseNet branch

Figure S5. Gensheimer head (24 months): ensemble stratifications obtained for the best-performing CNN and ViT configurations for the training and internal test data of the HECKTOR2021 cohort. Stratifications into low (blue) and high (orange) risk groups of LRC were obtained by using the median prediction on the training data as a cutoff. *P*-values of the log-rank test for differences between the strata's Kaplan-Meier curves are also provided. Transparently colored regions indicate 95% confidence intervals for the estimated survival functions.

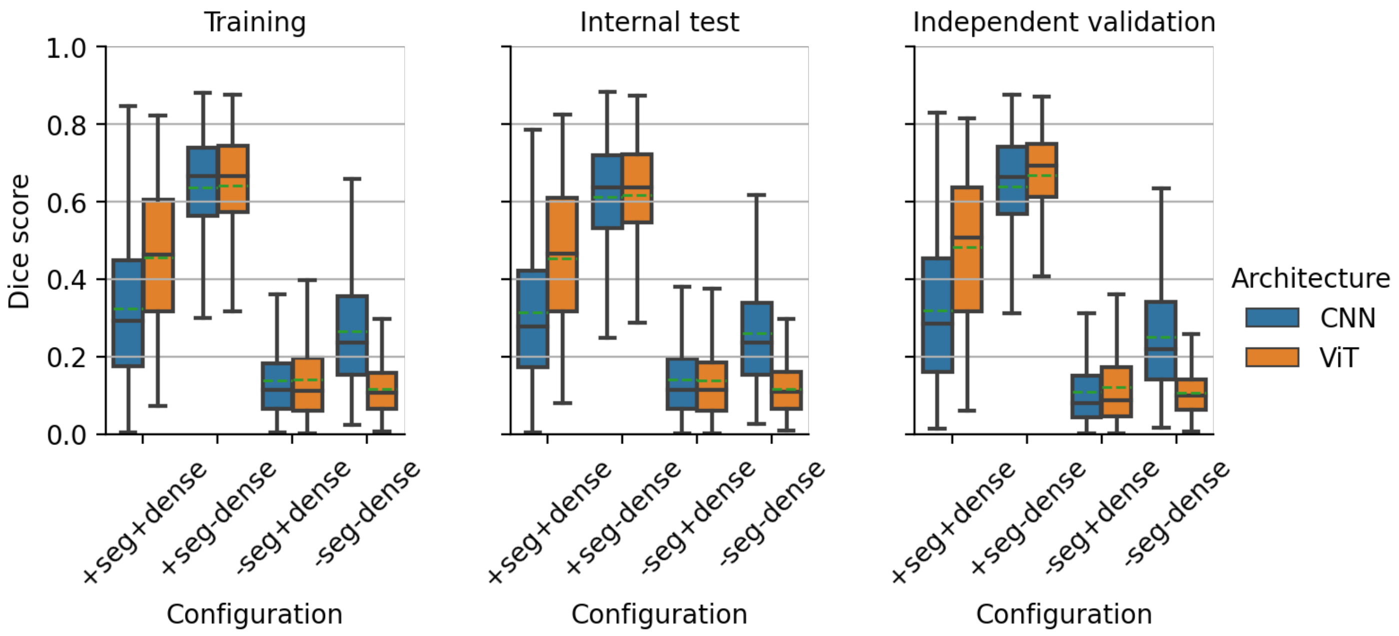

(a) DKTK cohort

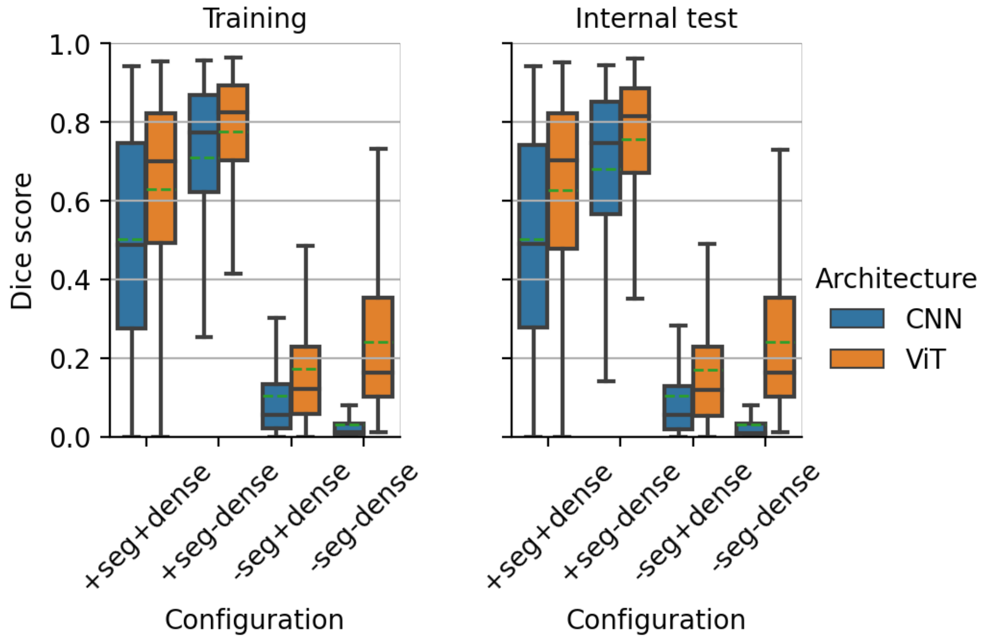

(b) HECKTOR2021 cohort

Figure S6. Cross-validation performance of the segmentation objective across all model configurations for multi-outcome models and both neural network architectures as measured by the Dice score. Green dashed lines denote distribution means.
